# OA14.04. A randomized controlled trial of spinal manipulation, medication or home exercise for acute and subacute neck pain

**DOI:** 10.1186/1472-6882-12-S1-O56

**Published:** 2012-06-12

**Authors:** G Bronfort, R Evans, A Anderson, K Svendsen, Y Bracha, R Grimm

**Affiliations:** 1 Northwestern Health Sciences University, Bloomington, USA; 2 Medical Pain Management, St. Louis Park, USA; 3 Cincinnati Children's Hospital, Cincinnati, USA; 4 Berman Center for Outcomes and Clinical Research, Minneapolis, USA

## Purpose

Mechanical neck pain is a common condition that affects almost three quarters of individuals at some point in their lives. Little research exists to guide the choice of therapy for acute and subacute neck pain. The purpose of this presentation is to present the results of a randomized clinical trial assessing the relative efficacy of spinal manipulation therapy (SMT), medication, and home exercise with advice (HEA) for acute and subacute neck pain.

## Methods

A total of 272 persons aged 18 to 65 years with a main complaint of nonspecific neck pain, 2 to 12 weeks duration were randomly assigned to 12 weeks of SMT, medication, or HEA. The primary outcome was participant-rated pain, measured at 2, 4, 8, 12, 26, and 52 weeks after randomization. Secondary measures were self-reported disability, global improvement, medication use, satisfaction, general health status and adverse events.

## Results

The SMT group had significantly less pain than the medication group after 8, 12, 26, and 52 weeks; HEA was superior to medication at 26 weeks. There were no differences in pain between SMT and HEA at any point. Results for most of the secondary outcomes were similar to those of the primary outcome.

## Conclusion

For individuals with acute and subacute neck pain, SMT was more effective than medication in both the short and long term. However, a few instructional sessions of HEA resulted in similar outcomes at most time points. (ClinicalTrials.gov registration number: NCT00029770)

